# Histopathological and prognostic significance of the expression of sex hormone receptors in bladder cancer: A meta-analysis of immunohistochemical studies

**DOI:** 10.1371/journal.pone.0174746

**Published:** 2017-03-31

**Authors:** Hiroki Ide, Satoshi Inoue, Hiroshi Miyamoto

**Affiliations:** 1 Department of Pathology, Johns Hopkins University School of Medicine, Baltimore, Maryland, United States of America; 2 The Brady Urological Institute, Johns Hopkins University School of Medicine, Baltimore, Maryland, United States of America; 3 Department of Pathology & Laboratory Medicine, University of Rochester Medical Center, Rochester, New York, United States of America; 4 The Wilmot Cancer Institute, University of Rochester Medical Center, Rochester, New York, United States of America; 5 Department Urology, University of Rochester Medical Center, Rochester, New York, United States of America; Centro Nacional de Investigaciones Oncologicas, SPAIN

## Abstract

**Objective:**

Emerging preclinical evidence suggests the involvement of sex hormones and their receptor signals in the development and progression of bladder cancer. Meanwhile, previous studies have demonstrated conflicting results on the relationship between the status of sex hormone receptors in urothelial tumors and histopathological characteristics of the tumors or patient outcomes. We therefore conducted this meta-analysis to assess the clinicopathological impact of the expression of androgen receptor (AR) and estrogen receptors (ERs) in bladder cancer.

**Methods:**

A comprehensive literature search in databases (*i*.*e*. PubMed, Web of Science, Cochrane) was performed for all immunohistochemical studies stained for AR, ERα, and/or ERβ in surgically resected bladder cancer specimens and analyzed for patient outcomes. We selected eligible studies in accordance with the PRISMA guidelines and analyzed data using R software.

**Results:**

A total of 2,049 patients from 13 retrospective studies were included in this meta-analysis. The difference in ERα expression between non-tumors and tumors was significant [odds ratio (OR) = 0.412; *P*<0.001], while those of AR (OR = 3.256; *P* = 0.336) or ERβ (OR = 0.580; *P* = 0.674) were not statistically significant. AR positivity in tumors was strongly correlated with gender (male *vs*. female: OR = 0.658; *P* = 0.027) or tumor grade (low-grade *vs*. high-grade: OR = 0.575; *P*<0.001). ERβ positive rates were significantly higher in high-grade (OR = 2.169; *P*<0.001) and muscle-invasive (OR = 3.104; *P*<0.001) tumors than in low-grade and non-muscle-invasive tumors, respectively. Survival analysis in patients with non-muscle-invasive bladder cancer revealed associations between AR expression and better recurrence-free survival [hazard ration (HR) = 0.593; *P* = 0.006) as well as between ERβ expression and worse recurrence-free (HR = 1.573; *P* = 0.013) or progression-free (HR = 4.148; *P* = 0.089) survivals.

**Conclusions:**

These data suggest down-regulation of ERα expression in bladder tumors, compared with non-neoplastic urothelial tissues. AR or ERβ expression was down- or up-regulated, respectively, in high-grade and/or muscle-invasive bladder cancers. Moreover, immunohistochemistry of AR/ERβ in surgical specimens may serve as prognosticators in patients with non-muscle-invasive bladder tumor.

## Introduction

Urinary bladder cancer is one of the most frequently diagnosed neoplasms, with an estimated 429,800 new cases and 165,100 deaths occurred in 2012 worldwide [1]. Although patients initially with non-muscle-invasive (NMI) tumor generally display favorable prognosis, they, especially those with high-grade tumor, have a relatively high risk of tumor recurrence with progression to muscle invasion after transurethral resection even with currently available intravesical pharmacotherapy. On the other hand, those with muscle-invasive (MI) tumor often develop disease progression or metastasis despite undergoing more aggressive treatment modalities, such as radical cystectomy with or without neoadjuvant or adjuvant systemic chemotherapy. Therefore, identification of key molecules involving bladder cancer outgrowth is urgently required, which may successively provide novel tumor markers that predict the prognosis as well as novel targeted therapy in patients with bladder cancer.

Various epidemiological and clinical studies have demonstrated that men have a substantially higher risk of bladder cancer, while women tend to have more aggressive tumors [1–7]. These observations have prompted research on sex hormone receptors, such as androgen receptor (AR) and estrogen receptors (ERs), in bladder cancer [8–10]. Indeed, preclinical evidence has suggested a critical role of AR/ERs in the development and progression of urothelial cancer arising in the upper and lower urinary tracts. Specifically, androgens have been shown to promote bladder tumorigenesis, as well as bladder cancer cell proliferation, migration, and invasion, via the AR pathway [11–22]. Non-androgen-mediated AR activation in bladder cancer cells [23] and androgen-induced tumorigenesis via the non-AR pathway [14] have also been documented. Moreover, AR activation has been correlated with resistance to chemotherapy in bladder cancer cells [24, 25]. In contrast, estrogens likely exert both stimulatory and inhibitory actions on bladder cancer outgrowth, which may be cell-specific and/or dependent on the functional activity of ERα and ERβ [11, 12, 26–32].

Meanwhile, conflicting data as to the expression of sex hormone receptors in urothelial tumors of the lower urinary tract, and its associations with tumor grade/stage or patient outcomes have been reported [33]. Therefore, in the current study using a meta-analysis, we aimed to determine the expression status of AR, ERα, and ERβ immunohistochemically detected in different grades or stages of bladder cancers and its potential role as prognosticators.

## Materials and methods

### Search strategy

A systematic literature search and subsequent quantitative analysis were planned, conducted, and reported according to the Preferred Reporting Items for Systematic Reviews and Meta-Analyses (PRISMA) guidelines [34] (S1 Fig, S1 Table). We performed a computerized bibliographic search of the PubMed, Web of Science, and Cochrane library in February 2017 for publications after 2004 in order to find the articles demonstrating the results of immunohistochemistry (IHC) of sex hormone receptors (*i*.*e*. AR, ERα, and/or ERβ) in bladder cancer tissue specimens obtained by transurethral resection or cystectomy from patients who did not receive radiotherapy or systemic chemotherapy preoperatively. The search strategy included the following keywords combined: 1) “androgen receptor” and “bladder cancer”; 2) “androgen receptor” and “urothelial carcinoma”; 3) “estrogen receptor” and “bladder cancer”; and 4) “estrogen receptor” and “urothelial carcinoma”. We selected only studies published in English in peer reviewed journals, while the results from letters and abstracts for meetings were excluded.

### Selection criteria

Eligible studies in this meta-analysis included those showing pathological and/or prognostic information in bladder cancer patients stratified with the expression of AR, ERα, or ERβ determined by IHC. Specifically, these studies compared the positivity of these receptors between non-tumor/normal bladder and tumor, low and high grades, and/or NMI and MI, or assessed prognostic significance of their expression [*e*.*g*. recurrence-free survival (RFS) or progression-free survival (PFS) in patients with NMI bladder cancer]. In the case of multiple publications from the same institution potentially with identical or overlapping patient cohorts, the most informative or the most complete study was used for our analysis. There was no restriction on geographical location of the studies.

### Data extraction

We recorded the following information about each eligible study: first author’s name; journal; year of publication; and number of patients analyzed. We also recorded available data on positivity of sex hormone receptors in non-tumor versus tumor, male versus female, low versus high grades, low versus high stages, RFS, and PFS (Table 1), as well as odds ratio (OR), hazard ratio (HR), and 95% confidence interval (CI). Data of multivariate analysis were included in the present study; if these data were not available, then univariate analysis data were used.

**Table 1:** Eligible immunohistochemical studies assessing the expression of sex hormone receptors in bladder tumors.

Author, year [reference]	Receptor	Patients (N)	Non-tumor (non-neoplastic urothelium) vs Tumor			Gender			Tumor grade			Tumor stage			Survival analysis (NMI)
			Non-tumor	Tumor	*P* value	Male	Female	*P* value	LG	HG	*P* value	NMI	MI	*P* value	
Boorjian, 2004 [38]	AR	49	86%	53%	0.001	61%	31%	0.104	89%	49%	0.055	75%	21%	0.002	NA
Boorjian, 2009 [39]	AR	55	NA	44%	0.06*	NA	NA	NA	NA	NA	NA	59%	33%	0.095	NA
Kauffman, 2011 [40]	AR	59	84%	51%	<0.001	NA	NA	NA	NA	NA	NA	NA	NA	NA	NA
Mir, 2011 [41]	AR	472	NA	13%	NA	14%	8%	0.159	12%	13%	1.000	9%	15%	0.086	NA
Tuygun, 2011 [42]	AR	139	0%	51%	<0.001	53%	41%	0.356	64%	37%	0.002	60%	21%	<0.001	RFS/PFS
Miyamoto, 2012 [36]	AR	188	80%	42%	<0.001	42%	43%	1.000	55%	36%	0.023	51%	33%	0.018	RFS/PFS
Jing, 2014 [20]	AR	58	NA	53%	NA	57%	43%	0.540	55%	50%	0.781	49%	69%	0.225	NA
Mashhadi, 2014 [43]	AR	120	0%	22%	<0.001	NA	NA	NA	NA	NA	<0.001*	NA	NA	<0.001*	NA
Nam, 2014 [44]	AR	169	NA	37%	NA	38%	31%	0.515	39%	33%	0.485	43% (Ta)	NA	0.048	RFS/PFS
												30% (T1)		(Ta vs T1)	
Miyamoto, 2012 [36]	ERα	188	50%	27%	<0.001	28%	25%	0.842	38%	23%	0.048	35%	19%	0.014	NS
Mashhadi, 2014 [43]	ERα	120	2%	3%	0.671	NA	NA	NA	NA	NA	NA	NA	NA	NA	NA
Croft, 2005 [45]	ERβ	92	NA	22%	NA	NA	NA	NA	12%	33%	0.021	NA	NA	NA	NA
Shen, 2006 [46]	ERβ	224	NA	63%	NA	NA	NA	NA	58%	70%	0.085	54%	80%	<0.001	NA
Kontos, 2010 [47]	ERβ	111	93%	76%	0.041	NA	NA	NA	NA	NA	NA	NA	54%	NA	NA
Tuygun, 2011 [42]	ERβ	139	7%	30%	<0.001	33%	23%	0.455	22%	31%	0.253	24%	36%	0.177	RFS/PFS
Miyamoto, 2012 [36]	ERβ	188	89%	49%	<0.001	53%	38%	0.109	29%	58%	<0.001	34%	67%	<0.001	RFS/PFS
Nam, 2014 [44]	ERβ	169	NA	31%	NA	31%	31%	1.000	27%	41%	0.098	22% (Ta)	NA	0.004	RFS/PFS
												42% (T1)		(Ta vs T1)	
Tan, 2015 [48]	ERβ	313	NA	100%	NA	NA	NA	NA	100%	100%	NS	100%	100%	NS	NA

AR = androgen receptor, ER = estrogen receptor, NA = not analyzed, M = male, F = female, LG = low-grade, HG = high-grade, NMI = non-muscle-invasive, MI = muscle-invasive, RFS = recurrence-free survival, PFS = progression-free survival, NS = not significant.

*Original data

### Quality assessment

Quality assessment was performed in each eligible study, using the Newcastle-Ottawa Scale (NOS) (ranging from 0 to 9). This tool has been developed to assess the quality of non-randomized studies to incorporate quality assessments in the interpretation of meta-analysis [35]. The NOS scores of 1–3, 4–6, and 7–9 were defined as low-, intermediate-, and high-quality studies, respectively.

### Statistical analysis

All analyses were carried out using R software (version 3.1.0). ORs and their 95% CIs were calculated based on the numbers from the studies to estimate the association between the expression of sex hormone receptors and pathological features of the tumors. Similarly, the pooled HR for RFS or PFS from published data was calculated by fixed and random-effects models in a multivariate setting. Original data for a study a senior author of the current study previously reported [36] were also used for calculating HRs and 95% CIs. Heterogeneity between studies was assessed by the Cochran’s Q test and *I*^*2*^ index, as previously described [37]. When heterogeneity among the studies was observed, we showed only the estimates of random-effects in the text. Publication bias was evaluated using Begg’s test. A *P* value of <0.05 was considered statistically significant.

## Results

### Search results and characteristics of included studies

**Fig 1:**
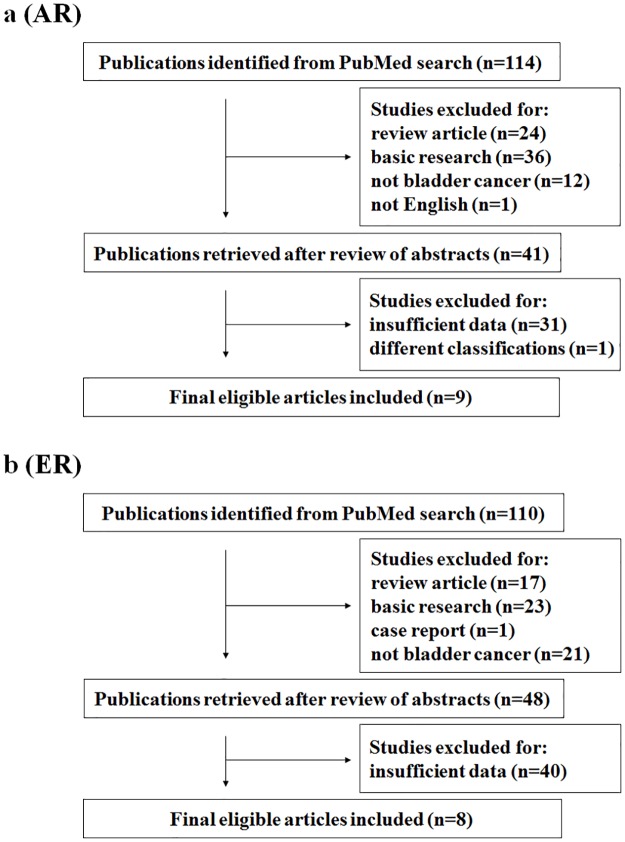
Flowchart of literature search and selection process. (a) AR and (b) ER.

We identified a total of 114 (AR) and 110 (ER) articles published between 2004 and 2017 by the primary computerized literature search. However, 73 (AR) and 62 (ER) were excluded because they were review articles, articles describing only the results derived from cell lines or animals, articles in non-bladder cancer, or articles written in non-English. The abstracts of the remainder of the articles were reviewed in detail, and 32 (AR) and 40 (ER) were further excluded due to no sufficient data or a different classification from other studies. Finally, 9 and 8 articles regarding AR and ER, respectively, were identified as eligible studies for this meta-analysis (Fig 1).

Four of the articles demonstrated the results of multiple receptors, and 13 eligible studies [20, 36, 38–48] involving a total of 2,049 bladder cancer patients were thus analyzed. The median sample size in these studies was 120 patients (range, 49–472). The relationship between pathological features (Table 2) or prognosis (Table 3) and each receptor expression had been individually assessed in 9 (AR), 2 (ERα), or 7 (ERβ) studies (Table 1).

**Table 2:** Meta-analysis between sex steroid hormone receptor expression and clinicopathological features of bladder cancers.

Stratification	Receptor	No. of Study [reference]	Patients (N)	Pooled OR (95% CI)			Heterogeneity	
				Fixed	Random	*P* value	*I*^*2*^ (%)	*P* value
Non-tumor vs Tumor	AR	5	555	1.138	3.256	0.336	95.7	<0.001
		[36,38,40,42,43]		(0.867–1.495)	(0.295–35.998)			
	ERα	2	308	0.412	0.605	<0.001	59.8	0.115
		[36,43]		(0.265–0.642)	(0.153–2.390)			
	ERβ	3	438	0.475	0.580	0.674	95.2	<0.001
		[36,42,47]		(0.327–0.691)	(0.046–7.370)			
Gender (male vs female)	AR	6	1075	0.658	0.664	0.027	0	0.653
		[20,36,38,41,42,44]		(0.454–0.954)	(0.457–0.967)			
	ERβ	3	496	0.660	0.662	0.101	0	0.559
		[36,42,44]		(0.401–1.085)	(0.402–1.088)			
Tumor grade (LG vs HG)	AR	6	1075	0.575	0.577	<0.001	43.9	0.113
		[20,36,38,41,43,44]		(0.421–0.785)	(0.367–0.908)			
	ERβ	6	1125	2.169	2.163	<0.001	0	0.453
		[36,42,44,45,46,48]		(1.583–2.971)	(1.547–3.023)			
Tumor stage (NMI vs MI)	AR	5	912	0.666	0.649	0.356	82.1	<0.001
		[20,36,39,41,42]		(0.476–0.930)	(0.260–1.625)			
	ERβ	4	864	3.104	3.077	<0.001	0	0.389
		[36,42,46, 48]		(2.081–4.631)	(2.055–4.608)			

AR = androgen receptor, ER = estrogen receptor, OR = odds ratio, CI = confidence interval, LG = low-grade, HG = high-grade, NMI = non-muscle-invasive, MI = muscle-invasive.

**Table 3:** Meta-analysis between sex hormone receptor expression in superficial bladder cancer and patient outcomes.

Survival	Receptor	No. of Study [reference]	Patients (N)	Pooled HR (95% CI)			Heterogeneity	
				Fixed	Random	*P* value	*I*^*2*^ (%)	*P* value
RFS	AR	3	496	0.593	0.598	0.006	19.4	0.289
		[36,42,44]		(0.408–0.860)	(0.393–0.911)			
	ERβ	3	496	1.573	1.573	0.013	0	0.553
		[36,42,44]		(1.102–2.247)	(1.102–2.247)			
PFS	AR	2	327	0.533	0.533	0.223	0	0.425
		[36,42]		(0.194–1.465)	(0.194–1.465)			
	ERβ	3	496	2.236	4.148	0.089	78.2	0.010
		[36,42,44]		(1.189–4.205)	(0.803–21.411)			

RFS = recurrence-free survival, PFS = progression-free survival, AR = androgen receptor, ER = estrogen receptor, HR = hazard ratio, CI = confidence interval.

Quality assessment using the NOS was performed in these 13 studies included in this meta-analysis. The NOSs were 6 or higher (S2 Fig), indicating that the quality of the studies was acceptable. In addition, the funnel plots via Begg’s test showed no significant bias across publications regarding differences in AR/ERβ expression in normal *vs*. tumor samples, patient genders, tumor grades/stages, or RFS/PFS rates (S3 Fig).

### AR

There were no statistically significant differences in AR expression between non-tumor and tumor (*P* = 0.336), non-tumor and NMI tumor (*P* = 0.664), or non-tumor and MI tumor (*P* = 0.515), as well as between NMI and MI tumors (*P* = 0.356). However, AR expression was significantly down-regulated in female tumors compared with male tumors (OR = 0.658; 95% CI = 0.454–0.954; *P* = 0.027) as well as in high-grade tumors compared with low-grade tumors (OR = 0.575; 95% CI = 0.421–0.785; *P* < 0.001). In NMI tumors, there was also a significant difference in AR positivity between low-grade and high-grade (OR = 0.457; 95% CI = 0.272–0.768; *P* = 0.003). Significant heterogeneity existed in the meta-analysis of the association of AR expression with non-tumor/tumor (*I*^2^ = 95.7%; *P* < 0.001) or tumor stage (*I*^2^ = 82.1%; *P* < 0.001), but not with gender (*P* = 0.653) or tumor grade (*P* = 0.113).

HRs of AR positivity for RFS and PFS were available in 3 and 2 studies accounting for 496 and 327 patients with NMI bladder tumor, respectively. AR expression was significantly associated with better RFS (HR = 0.593; 95% CI = 0.408–0.860; *P* = 0.006), but not PFS (*P* = 0.223). No significant heterogeneity of association between AR expression and RFS (*P* = 0.289) or PFS (*P* = 0.425) was identified. The association between AR expression and prognosis in patients with MI tumor could not be analyzed because only one study [36] demonstrated such data.

### ERα

ERα expression was significantly down-regulated in bladder tumors, compared with non-tumors (OR = 0.412; 95% CI = 0.265–0.642; *P* < 0.001), with exhibiting no significant heterogeneity (*P* = 0.115). We could not analyze the associations between ERα expression and gender, tumor grade/stage, or prognosis because only one study [36] demonstrated such data.

### ERβ

There was no statistically significant difference in ERβ expression between non-tumor and tumor (*P* = 0.674), non-tumor and NMI tumor (*P* = 0.612), or non-tumor and MI tumor (*P* = 0.663), as well as between male and female tumors (*P* = 0.101). However, ERβ expression was significantly up-regulated in high-grade tumors compared with low-grade tumors (OR = 2.169; 95% CI = 1.583–2.971; *P* < 0.001) as well as in MI tumors compared with NMI tumors (OR = 3.104; 95% CI = 2.081–4.631; *P* < 0.001). In a subgroup of NMI tumors, there was no significant difference in ERβ positivity between low-grade and high-grade (*P* = 0.989). Significant heterogeneity existed in the meta-analysis of the association of ERβ expression with non-tumor/tumor (*I*^2^ = 95.2%; *P* < 0.001), but not with gender (*P* = 0.559) or tumor grade (*P* = 0.453) or stage (*P* = 0.389).

HR of ERβ positivity for either RFS or PFS was available in 3 studies accounting for 496 patients with NMI bladder tumor. ERβ expression was significantly or marginally associated with worse RFS (HR = 1.573; 95% CI = 1.102–2.247; *P* = 0.013) or PFS (HR = 4.148; 95% CI = 0.803–21.411; *P* = 0.089), respectively. No significant heterogeneity of association of ERβ expression with RFS (*P* = 0.553) was seen, whereas that with PFS was significant (*I*^2^ = 78.2%; *P* = 0.010). The association between ERβ expression and prognosis in patients with MI tumor could not be analyzed because only one study [36] demonstrated such data.

## Discussion

IHC has detected AR protein signals in 13–55% of bladder or upper urinary tract urothelial tumors [19, 20, 36, 38–44, 49–51], which is significantly lower than the positive rates in normal/non-neoplastic urothelial tissues (58–86%) reported by most of respective comparative studies [36, 38, 40, 51]. However, at least three immunohistochemical studies have demonstrated no AR expression in normal urothelium [42, 43, 52]. Significant or insignificant down-regulation of AR expression in high-grade or MI urothelial carcinomas, compared with low-grade or NMI tumors, has also been found [19, 36, 38–40, 42, 44, 49–51]. However, several studies showed even slight increases in AR positivity in high-grade and/or MI tumors [20, 41, 51]. Furthermore, two studies each have suggested a considerable association of AR expression in bladder tumors with a higher risk of the progression of only MI disease [36] or both NMI and MI diseases [43] or a lower risk of the recurrence of NMI disease [42, 44], or no such strong association with the prognosis of patients with NMI or MI disease [40, 41].

Although *ERα* gene expression via a quantitative polymerase chain reaction (PCR) method has been confirmed in all the 10 tumors examined [29], ERα signals via IHC in tissue specimens have been detected only in a small subset (*e*.*g*. 1–5%) of bladder cancers in most of previous studies [46, 48, 53]. In contrast, our IHC showed that ERα was positive in 27% of bladder tumors [36] as well as in 18% of upper urinary tract tumors [51]. Some of these immunohistochemical studies have also demonstrated elevated levels of ERα expression in non-neoplastic urothelial tissues, compared with bladder tumors [36, 51, 53], in contrast to the findings in the PCR analysis (*i*.*e*. 2.77-fold stronger expression in tumors than in matched normal tissues) [29], as well as in low-grade/NMI tumors, compared with high-grade/MI tumors [36, 53]. In accordance with the IHC data, three separate microarray cohorts of bladder tissues showed significantly lower levels of ERα gene expression in tumor than in normal [54–56]. However, none of the studies have shown prognostic significance of ERα expression in patients with urothelial tumor. Our recent study demonstrated that patients with pT3-4 upper urinary tract urothelial carcinoma negative for both ERα and progesterone receptor (PR) had a significantly lower risk of cancer-specific mortality, compared with those showing ERα and/or PR positivity [51].

The positive rates of ERβ expression in immunohistochemical studies in urothelial tumor specimens range from 22% to 100% [19, 36, 40, 42, 44–48, 51], which was significantly lower than those in non-neoplastic urothelial tissues in most of comparative studies [36, 40, 47, 51]. In addition, both significant or insignificant up-regulation [36, 42, 44–46] and down-regulation [47] of ERβ expression have been reported in higher grade/stage tumors. Strong associations of ERβ expression with both the risk of recurrence and/or progression of NMI tumors [36, 42, 44] or progression of MI tumors [36] and favorable prognosis in patients with NMI tumor [48] have also been documented.

The expression status of sex hormone receptors has thus been assessed in bladder cancer tissues, mainly using IHC, resulting in conflicting results regarding the rate of receptor positivity as well as the relationship between receptor expression and tumor aggressiveness. These included a discrepancy in, for instance, AR positivity between bladder cancer tissues obtained at different institutions but stained at one institution using the same antibody and staining protocol [36, 41]. In addition, the impact of AR/ERα/ERβ expression on patient outcomes as prognosticators remains controversial. As shown in breast tissues [57, 58], these varied data in IHC may be attributed to the differences in antibody specificity or staining strategy as well as tissue preparation including preservation in fixative. We therefore conducted this meta-analysis of previous studies demonstrating the expression of AR, ERα, and ERβ in bladder cancer specimens.

Our analysis for AR expression, showing significant heterogeneity in non-tumor/tumor and tumor stage, but not in tumor grade or prognosis, revealed its strong inverse association with tumor grade, as well as no significant differences between benign bladders versus tumors and between NMI versus MI tumors. Patients with AR-positive NMI tumor were also found to have a significantly lower risk for tumor recurrence, compared to those with AR-negative NMI, but not for disease progression. Interestingly, while each previous study has demonstrated no statistically significant difference in AR positivity between tumors from male versus female patients [20, 36, 38, 41, 42, 44], the meta-analysis shows significant down-regulation of AR expression in female tumors. The rate of ERα positivity was found to be significantly lower in bladder tumors than in non-tumors. However, no further analysis could be performed due to insufficient number of studies. While ERβ positivity was not significantly different between non-tumors and tumors with significant heterogeneity, as well as between male and female tumors without significant heterogeneity, in the analysis, significant up-regulation was seen in high-grade or MI tumors, compared with low-grade or NMI tumors. Patients with ERβ-positive NMI tumor were also found to have a significantly higher risk for tumor recurrence, compared to those with ERβ-negative NMI tumor. Similarly, there was a tendency to correlate between ERβ expression and disease progression in patients with NMI tumor.

The NOSs of previous non-randomized studies included in this meta-analysis were found to be 6–8, indicating high-quality of each study. In addition, Begg’s test revealed no statistical significance, suggesting that this meta-analysis was not biased. Nonetheless, in this study, there are several limitations that need to be carefully considered when interrupting the results. First, as stated above, significant heterogeneity among included studies existed. Second, the potential risk bias was a concern. Because positive results were more likely to be published than negative ones, the meta-analyses based on published data might overestimate clinical significance of the expression status of sex hormone receptors in bladder cancer. Third, there were differences in staining protocol, including antibody, as well as scoring of the stains, used in previous studies, which might have resulted in diverse expression patterns. Finally, all included in this meta-analysis were retrospective studies often susceptible to selection bias.

In summary, we assessed the expression status of AR, ERα, and ERβ in bladder cancers and its potential role as prognosticators. We found some differences in receptor positivity between non-neoplastic bladder tissue and bladder cancer as well as tumor grades or stages. AR or ERβ expression was also found to correlate with tumor recurrence or progression, respectively. These findings support previous preclinical data indicating the involvement of sex hormone receptor signals in urothelial carcinogenesis and cancer progression. Moreover, the rate of AR positivity was significant higher in male tumors than in female tumors, while none of previous studies showed such a statistically significant difference. Because the number of the studies included in the current meta-analysis for each receptor is relatively small, we may need to accumulate more data to re-evaluate the significance of AR/ERα/ERβ expression in bladder cancer outgrowth.

## Supporting information

S1 Fig The PRISMA flow diagram of the meta-analysis. (DOC)

S2 Fig Quality assessment of eligible studies with Newcastle-Ottawa Scale (NOS) on 3 levels: Selection, comparability, and outcome. (TIF)

S3 Fig Funnel plots for publication bias test from 3 or more studies in each category. (TIF)

S1 Table The PRISMA checklist of the meta-analysis. (DOC)

## References

[1] Torre LA, Bray F, Siegel RL, Ferlay J, Lortet-Tieulent J, Jemal A. Global cancer statistics, 2012. CA Cancer J Clin 2015; 65: 87–108. 10.3322/caac.21262 25651787

[2] Scosyrev E, Trivedi D, Messing E. Female bladder cancer: incidence, treatment, and outcome. Curr Opin Urol 2010; 20: 404–408. 10.1097/MOU.0b013e32833c7a9b 20592613

[3] Fajkovic H, Halpern JA, Cha EK, Bahadori A, Chromecki TF, Karakiewicz PI, et al Impact of gender on bladder cancer incidence, staging, and prognosis. World J Urol 2011; 29: 457–463. 10.1007/s00345-011-0709-9 21656173

[4] Kluth LA, Fajkovic H, Xylinas E, Crivelli JJ, Passoni N, Rouprêt M, et al Female gender is associated with higher risk of disease recurrence in patients with primary T1 high-grade urothelial carcinoma of the bladder. World J Urol 2013; 31: 1029–1036. 10.1007/s00345-012-0996-9 23196773

[5] Lucca I, Fajkovic H, Klatte T. Sex steroids and gender differences in nonmuscle invasive bladder cancer. Curr Opin Urol 2014; 24: 500–505. 10.1097/MOU.0000000000000092 24978392

[6] Schmid M, Shariat SF, Soave A, Engel O, Fisch M, Rink M. Contemporary gender-specific outcomes in Germany after radical cystectomy for bladder cancer. Curr Urol Rep 2014; 15: 409 10.1007/s11934-014-0409-2 24756451

[7] Siegel RL, Miller KD, Jemal A. Cancer statistics, 2016. CA Cancer J Clin 2016; 66: 7–30. 10.3322/caac.21332 26742998

[8] Miyamoto H, Zheng Y, Izumi K. Nuclear hormone receptor signals as new therapeutic targets for urothelial carcinoma. Curr Cancer Drug Tar 2012; 12: 14–22. 10.2174/156800912798888965 22111835

[9] Li Y, Izumi K, Miyamoto H. The role of the androgen receptor in the development and progression of bladder cancer. Jpn J Clin Oncol 2012; 42: 569–577. 10.1093/jjco/hys072 22593639

[10] Hsu I, Vitkus S, Da J, Yeh S. Role of oestrogen receptors in bladder cancer development. Nat Rev Urol 2013; 10: 317–326. 10.1038/nrurol.2013.53 23588401

[11] Okajima E, Hiramatsu T, Iriya K, Ijuin M, Matsushima S. Effects of sex hormones on development of urinary bladder tumours in rats induced by N-butyl-N-(4-hydroxybutyl) nitrosamine. Urol Res 1975; 3: 73–79. 1162802 10.1007/BF00256185

[12] Reid LM, Leav I, Kwan PW, Russell P, Merk FB. Characterization of a human, sex steroid-responsive transitional cell carcinoma maintained as a tumor line (R198) in athymic nude mice. Cancer Res 1984; 44: 4560–4573. 6467211

[13] Imada S, Akaza H, Ami Y, Koiso K, Ideyama Y, Takenaka T. Promoting effects and mechanisms of action of androgen in bladder carcinogenesis in male rats. Eur Urol 1997; 31: 360–364. 9129932 10.1159/000474484

[14] Miyamoto H, Yang Z, Chen YT, Ishiguro H, Uemura H, Kubota Y, et al Promotion of bladder cancer development and progression by androgen receptor signals. J Natl Cancer Inst 2007; 99: 558–568. 10.1093/jnci/djk113 17406000

[15] Johnson AM, O'Connell MJ, Miyamoto H, Huang J, Yao JL, Messing EM, et al Androgenic dependence of exophytic tumor growth in a transgenic mouse model of bladder cancer: a role for thrombospondin-1. BMC Urol 2008; 8: 7 10.1186/1471-2490-8-7 18433501 PMC2374790

[16] Wu JT, Han BM, Yu SQ, Wang HP, Xia SJ. Androgen receptor is a potential therapeutic target for bladder cancer. Urology 2010; 75: 820–827. 10.1016/j.urology.2009.10.041 20083299

[17] Zheng Y, Izumi K, Yao JL, Miyamoto H. Dihydrotestosterone upregulates the expression of epidermal growth factor receptor and ERBB2 in androgen receptor-positive bladder cancer cells. Endocr Relat Cancer 2011; 18: 451–464. 10.1530/ERC-11-0010 21613411

[18] Overdevest JB, Knubel KH, Duex JE, Thomas S, Nitz MD, Harding MA, et al CD24 expression is important in male urothelial tumorigenesis and metastasis in mice and is androgen regulated. Proc Natl Acad Sci USA 2012; 109: E3588–E3596. 10.1073/pnas.1113960109 23012401 PMC3529071

[19] Shyr CR, Chen CC, Hsieh TF, Chang CH, Ma WL, Yeh S, et al The expression and actions of androgen receptor in upper urinary tract urothelial carcinoma (UUTUC) tissues and the primary cultured cells. Endocrine 2013; 43: 191–199. 10.1007/s12020-012-9762-4 22851332

[20] Jing Y, Cui D, Guo W, Jiang J, Jiang B, Lu Y, et al Activated androgen receptor promotes bladder cancer metastasis via Slug mediated epithelial-mesenchymal transition. Cancer Lett 2014; 348: 135–145. 10.1016/j.canlet.2014.03.018 24662746

[21] Li Y, Ishiguro H, Kawahara T, Miyamoto Y, Izumi K, Miyamoto H. GATA3 in the urinary bladder: suppression of neoplastic transformation and down-regulation by androgens. Am J Cancer Res 2014; 4: 461–473. 25232488 PMC4163611

[22] Kawahara T, Shareef HK, Aljarah AK, Ide H, Li Y, Kashiwagi E, et al ELK1 is up-regulated by androgen in bladder cancer cells and promotes tumor progression. Oncotarget 2015; 6: 29860–29876. 10.18632/oncotarget.5007 26342199 PMC4745768

[23] Izumi K, Zheng Y, Li Y, Zaengle J, Miyamoto H. Epidermal growth factor induces bladder cancer cell proliferation through activation of the androgen receptor. Int J Oncol 2012; 41: 1587–1592. 10.3892/ijo.2012.1593 22922989 PMC3583640

[24] Shiota M, Takeuchi A, Yokomizo A, Kashiwagi E, Tatsugami K, Kuroiwa K, et al Androgen receptor signaling regulates cell growth and vulnerability to doxorubicin in bladder cancer. J Urol 2012; 188: 276–286. 10.1016/j.juro.2012.02.2554 22608749

[25] Kashiwagi E, Ide H, Inoue S, Kawahara T, Zheng Y, Reis LO, et al Androgen receptor activity modulates responses to cisplatin treatment in bladder cancer. Oncotarget 2016; 7: 49169–49179. 10.18632/oncotarget.9994 27322140 PMC5226499

[26] Kim HT, Kim BC, Kim IY, Mamura M, Seong DH, Jang JJ, et al Raloxifene, a mixed estrogen agonist/antagonist, induces apoptosis through cleavage of BAD in TSU-PR1 human cancer cells. J Biol Chem 2002; 277: 32510–32515. 10.1074/jbc.M202852200 12084714

[27] Waalkes MP, Liu J, Ward JM, Powell DA, Diwan BA. Urogenital carcinogenesis in female CD1 mice induced by in utero arsenic exposure is exacerbated by postnatal diethylstilbestrol treatment. Cancer Res 2006; 66: 1337–1345. 10.1158/0008-5472.CAN-05-3530 16452187

[28] Sonpavde G, Okuno N, Weiss H, Yu J, Shen SS, Younes M, et al Efficacy of selective estrogen receptor modulators in nude mice bearing human transitional cell carcinoma. Urology 2007; 69: 1221–1226. 10.1016/j.urology.2007.02.041 17572228

[29] Teng J, Wang ZY, Jarrard DF, Bjorling DE. Roles of estrogen receptor α and β in modulating urothelial cell proliferation. Endocr Relat Cancer 2008; 15: 351–364. 10.1677/erc.1.01255 18310301 PMC3513362

[30] Hsu I, Chuang KL, Slavin S, Da J, Lim WX, Pang ST, et al Suppression of ERβ signaling via ERβ knockout or antagonist protects against bladder cancer development. Carcinogenesis 2014; 35: 651–661. 10.1093/carcin/bgt348 24148819

[31] Hsu I, Yeh CR, Slavin S, Miyamoto H, Netto GJ, Tsai YC, et al Estrogen receptor alpha prevents bladder cancer via INPP4B inhibited akt pathway *in vitro* and *in vivo*. Oncotarget 2014; 5: 7917–7935. 10.18632/oncotarget.1421 25277204 PMC4202170

[32] Yeh CR, Hsu I, Song W, Chang H, Miyamoto H, Xiao GQ, et al Fibroblast ERα promotes bladder cancer invasion via increasing the CCL1 and IL-6 signals in the tumor microenvironment. Am J Cancer Res 2105; 5: 1146–1157. PMC4449442 26045993

[33] Ide H, Miyamoto H. Steroid hormone receptor signals as prognosticators for urothelial tumor. Dis Markers 2015; 2015: 840640 10.1155/2015/840640 26770009 PMC4685115

[34] Moher D, Liberati A, Tetzlaff J, Altman DG. Preferred reporting items for systematic reviews and meta-analyses: the PRISMA statement. BMJ 2009; 339: b2535 10.1136/bmj.b2535 19622551 PMC2714657

[35] Lo CK, Mertz D, Loeb M. Newcastle-Ottawa Scale: comparing reviewers' to authors' assessments. BMC Med Res Methodol 2014; 14: 45 10.1186/1471-2288-14-45 24690082 PMC4021422

[36] Miyamoto H, Yao JL, Chaux A, Zheng Y, Hsu I, Izumi K, et al Expression of androgen and oestrogen receptors and its prognostic significance in urothelial neoplasm of the urinary bladder. BJU Int 2012; 109: 1716–1726. 10.1111/j.1464-410X.2011.10706.x 22221549

[37] Higgins JP, Thompson SG, Deeks JJ, Altman DG. Measuring inconsistency in meta-analyses. BMJ 2003; 327: 557–560. 10.1136/bmj.327.7414.557 12958120 PMC192859

[38] Boorjian S, Ugras S, Mongan NP, Gudas LJ, You X, Tickoo SK, et al Androgen receptor expression is inversely correlated with pathologic tumor stage in bladder cancer. Urology 2004; 64: 383–388. 10.1016/j.urology.2004.03.025 15302512

[39] Boorjian SA, Heemers HV, Frank I, Farmer SA, Schmidt LJ, Sebo TJ, et al Expression and significance of androgen receptor coactivators in urothelial carcinoma of the bladder. Endocr Relat Cancer 2009; 16: 123–137. 10.1677/ERC-08-0124 18845648 PMC2674368

[40] Kauffman EC, Robinson BD, Downes MJ, Powell LG, Lee MM, Scherr DS, et al Role of androgen receptor and associated lysine-demethylase coregulators, LSD1 and JMJD2A, in localized and advanced human bladder cancer. Mol Carcinogen 2011; 50: 931–944. 10.1002/mc.20758 PMC3315373 21400613

[41] Mir C, Shariat SF, van der Kwast TH, Ashfaq R, Lotan Y, Evans A, et al Loss of androgen receptor expression is not associated with pathological stage, grade, gender or outcome in bladder cancer: a large multi-institutional study. BJU Int 2011; 108: 24–30. 10.1111/j.1464-410X.2010.09834.x 21070579

[42] Tuygun C, Kankaya D, Imamoglu A, Sertcelik A, Zengin K, Oktay M, et al Sex-specific hormone receptors in urothelial carcinomas of the human urinary bladder: a comparative analysis of clinicopathological features and survival outcomes according to receptor expression. Urol Oncol 2011; 29: 43–51. 10.1016/j.urolonc.2009.01.033 19372057

[43] Mashhadi R, Pourmand G, Kosari F, Mehrsai A, Salem S, Pourmand MR, et al Role of steroid hormone receptors in formation and progression of bladder carcinoma: a case-control study. Urol J 2014; 11: 1968–1973. 25433476

[44] Nam JK, Park SW, Lee SD, Chung MK. Prognostic value of sex-hormone receptor expression in non-muscle-invasive bladder cancer. Yonsei Med J 2014; 55: 1214–1221. 10.3349/ymj.2014.55.5.1214 25048477 PMC4108804

[45] Croft PR, Lathrop SL, Feddersen RM, Joste NE. Estrogen receptor expression in papillary urothelial carcinoma of the bladder and ovarian transitional cell carcinoma. Arch Pathol Lab Med 2005; 129: 194–199. 10.1043/1543-2165(2005)129<194:EREIPU>2.0.CO;2 15679420

[46] Shen SS, Smith CL, Hsieh JT, Yu J, Kim IY, Jian W, et al Expression of estrogen receptors-α and -β in bladder cancer cell lines and human bladder tumor tissue. Cancer 2006; 106: 2610–2616. 10.1002/cncr.21945 16700038

[47] Kontos S, Kominea A, Melachrinou M, Balampani E, Sotiropoulou-Bonikou G. Inverse expression of estrogen receptor-β and nuclear factor-κB in urinary bladder carcinogenesis. Int J Urol 2010; 17: 801–809. 10.1111/j.1442-2042.2010.02603.x 20727050

[48] Tan W, Boorjian S, Advani P, Farmer S, Lohse C, Cheville J, et al The estrogen pathway: Estrogen receptor-α, progesterone receptor, and estrogen receptor-β expression in radical cystectomy urothelial cell carcinoma specimens. Clin Genitourin Cancer 2015; 13: 476–484. 10.1016/j.clgc.2015.04.001 25981333

[49] Rau KM, Chen YJ, Sun MT, Kang HY. Prognostic effects and regulation of activin A, maspin, and the androgen receptor in upper urinary tract urothelial carcinoma. Anticancer Res 2011 31: 1713–1720. 21617230

[50] Williams EM, Higgins JP, Sangoi AR, McKenney JK, Troxell ML. Androgen receptor immunohistochemistry in genitourinary neoplasms. Int Urol Nephrol 2015; 47: 81–85. 10.1007/s11255-014-0834-7 25218615

[51] Kashiwagi E, Fujita K, Yamaguchi S, Fushimi H, Ide H, Inoue S, et al Expression of steroid hormone receptors and its prognostic significance in urothelial carcinoma of the upper urinary tract. Cancer Biol Ther 2016; 17: 1188–1196. 10.1080/15384047.2016.1235667 27635763 PMC5137486

[52] Birtle AJ, Freeman A, Munson P. The androgen receptor revisited in urothelial carcinoma. Histopathology 2004; 45: 98–99. 10.1111/j.1365-2559.2004.01841.x 15228457

[53] Bolenz C, Lotan Y, Ashfaq R, Shariat SF. Estrogen and progesterone hormonal receptor expression in urothelial carcinoma of the bladder. Eur Urol 2009; 56: 1093–1095. 10.1016/j.eururo.2009.06.032 19596509

[54] Dyrskjøt L, Kruhøffer M, Thykjaer T, Marcussen N, Jensen JL, Møller K, et al Gene expression in the urinary bladder: a common carcinoma in situ gene expression signature exists disregarding histopathological classification. Cancer Res 2004; 64: 4040–4048. 10.1158/0008-5472.CAN-03-3620 15173019

[55] Sanchez-Carbayo M, Socci ND, Lozano J, Saint F, Cordon-Cardo C. Defining molecular profiles of poor outcome in patients with invasive bladder cancer using oligonucleotide microarrays. J Clin Oncol 2006; 24: 778–789. 10.1200/JCO.2005.03.2375 16432078

[56] Lee JS, Leem SH, Lee SY, Kim SC, Park ES, Kim SB, et al Expression signature of E2F1 and its associated genes predict superficial to invasive progression of bladder tumors. J Clin Oncol 2010; 28: 2660–2667. 10.1200/JCO.2009.25.0977 20421545

[57] Skliris GP, Parkes AT, Limer JL, Burdall SE, Carder PJ, Speirs V. Evaluation of seven oestrogen receptor β antibodies for immunohistochemistry, western blotting, and flow cytometry in human breast tissue. J Pathol 2002; 197: 155–162. 10.1002/path.1077 12015738

[58] Goldstein NS, Ferkowicz M, Odish E, Mani A, Hastah F. Minimum formalin fixation time for consistent estrogen receptor immunohistochemical staining of invasive breast carcinoma. Am J Clin Pathol 2003; 120: 86–92. 10.1309/QPHD-RB00-QXGM-UQ9N 12866377

