# Supplementary material for: Histopathological and prognostic significance of the expression of sex hormone receptors in bladder cancer: A meta-analysis of immunohistochemical studies
Source: PLoS One. 2017 Mar 31;12(3):e0174746. doi: 10.1371/journal.pone.0174746 (PMC5375178; doi:10.1371/journal.pone.0174746)
Supplement: S1 Fig — (DOC) [file pone.0174746.s001.doc]

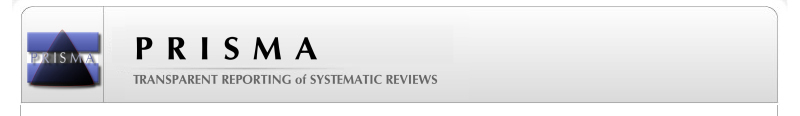
**PRISMA 2009 Flow Diagram**

**Screening**

**Included**

**Eligibility**

**Identification**

Records identified through database searching
(n = 200)

Additional records identified through other sources
(n = 0)

Records after duplicates removed
(n = 200)

Records screened
(n = 200)

Records excluded
(n = 187)

Full-text articles assessed for eligibility
(n = 13)

Full-text articles excluded, with reasons
(n = 0)

Studies included in qualitative synthesis
(n = 13)

Studies included in quantitative synthesis (meta-analysis)
(n = 13)
