# Supplementary figures and images for: Histopathological and prognostic significance of the expression of sex hormone receptors in bladder cancer: A meta-analysis of immunohistochemical studies
Source: PLoS One. 2017 Mar 31;12(3):e0174746. doi: 10.1371/journal.pone.0174746 (PMC5375178; doi:10.1371/journal.pone.0174746)

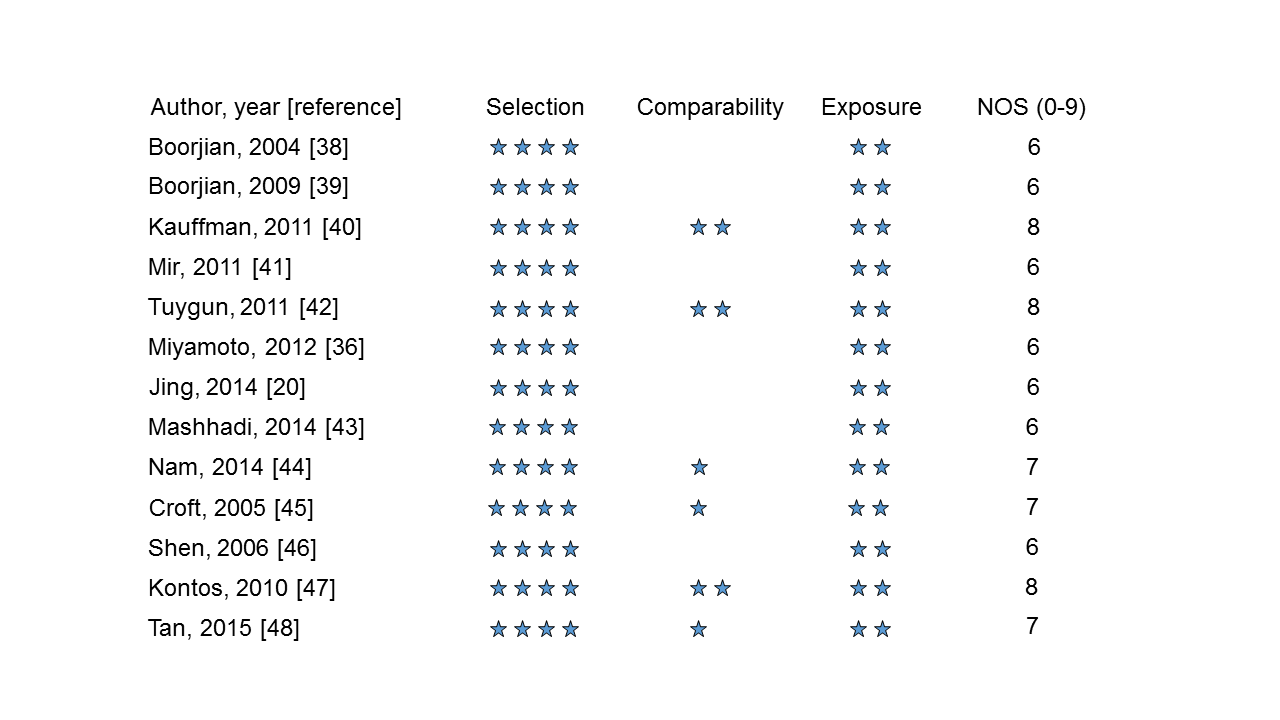

Supplement: S2 Fig — (TIF) [file pone.0174746.s002.tif]

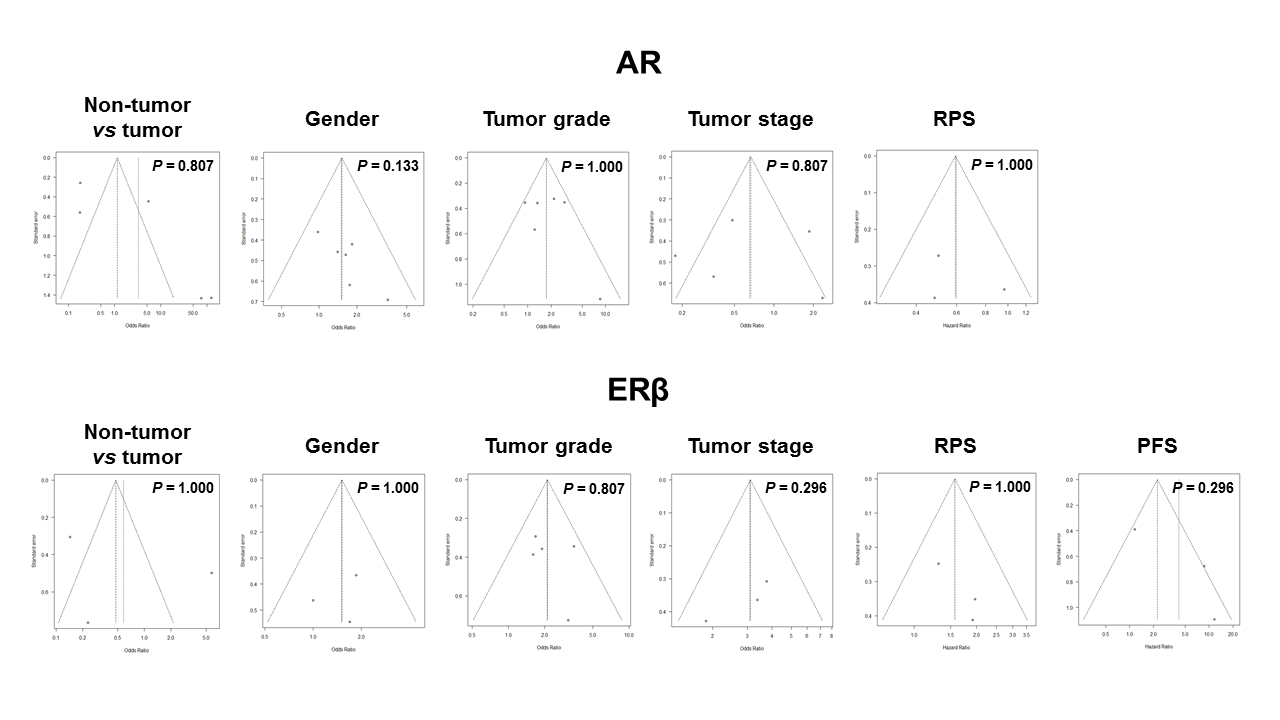

Supplement: S3 Fig — (TIF) [file pone.0174746.s003.tif]
